# Supplementary material for: Novel Competitive Fluorescence Sensing Platform for L-carnitine Based on Cationic Pillar[5]Arene Modified Gold Nanoparticles
Source: Sensors (Basel). 2018 Nov 14;18(11):3927. doi: 10.3390/s18113927 (PMC6263671; doi:10.3390/s18113927)
Supplement: Supplementary file 1 [file sensors-18-03927-s001.zip › sensors-384587-supplementary.pdf]

# Suppporting Information

## Novel competitive fluorescence sensing platform for L-carnitine based on cationic pillar[5]arene modified gold nanoparticles

Xiaoping Tan [a,*], Yang Yang [a], Shasha Luo [a], Zhong Zhang [a], Wenjie Zeng [a], Tingying Zhang [a], Fawu Su [b,*], Linzong Zhou [c,*]

[a] Key Lab of Inorganic Special Functional Materials, Chongqing Municipal Education Commission, School of Chemistry and Chemical Engineering, Yangtze Normal University, Fuling 408100, Chongqing, China

[b] State Key Laboratory for Conservation and Utilization of Bio-Resources in Yunnan, Yunnan Agricultural University, Kunming 650224, Yunnan, China

[c] School of geographical science and tourism management, Chuxiong Normal University, Chuxiong 675000, Yunnan, China

***S1 Molecular Docking***

The structures of CP5 and L-carnitine were constructed using AutoDockTools 1.5.6.[1] The CP5 crystal structure (ID: 4118702) was gained from Cambridge Crystallographic Data Centre (CCDC) with similar crystal structure. The AM1-BCC charges and GAFF force field were used. The optimized structures were used as starting structures. The sphgen module was applied to generate spheres surrounding the binding site. The Grid module of AutoDock 4.2.6 was employed to generate a grid file, which was used for scoring in the subsequent docking procedure. The flexible docking method was utilized to produce 1000 different conformational orientations for the guest molecule. The electrostatic interactions were calculated based on the grids. Finally, clustering analysis with an Amber14 was performed to obtain the best results.[2]

***S1. Reagents and methods***

1,4-Bis(2-hydroxyethoxy)benzene, dichloroethane, boron trifluoride diethyl etherate, carbon tetrabromide, triphenylphosphine, acetonitrile, paraformaldehyde, and trimethylamine were reagent grade and used as received. Solvents were either employed as purchased or dried according to procedures described in the literature. CP5 was synthesized according to the previous papers procedures.[3,4]

***S2. Synthesis of cationic pillar[5]arene (CP5)***

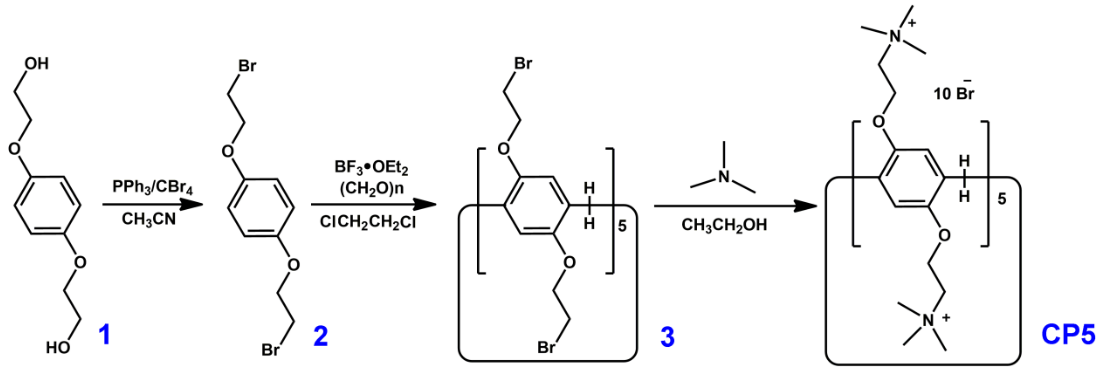


***Scheme S1.*** Synthetic route of **CP5**.

**Synthesis of 2:** A solution of **1** (5.0 g, 25.0 mmol) and triphenylphosphine (16.0 g, 60 mmol) in dry acetonitrile (200 mL) was cooled with an ice bath. Under vigorous stirring, carbon tetrabromide (20.0 g, 60 mmol) was slowly added. The mixture was stirred at room temperature for 4 hours. Then cold water (150 mL) was added to the

reaction mixture to give white precipitation. The precipitate was collected, washed with methanol/water (3:2, 3 × 100 mL), recrystallized from methanol, and dried under vacuum to afford **2** as white crystals (5.9 g, 91%). The $^{1}$H NMR spectrum of **2** is shown in Figure S1. $^{1}$H NMR (400 MHz, $CDCl_3$, rt) $\delta$ (ppm): 6.863 (s, 4H), 4.245 (t, $J$ = 5.6 Hz, 4H), 3.618 (t, $J$ = 6.4 Hz, 4H). The $^{13}$C NMR spectrum of **2** is shown in Figure S2. $^{13}$C NMR (100 MHz, $CDCl_3$, rt) δ (ppm): 152.81, 116.07, 68.69, 29.29.

**Synthesis of 3:** Boron trifluoride diethyl etherate ($BF_3{\cdot}OEt_2$, 3.26 g, 23.0 mmol) was added to the mixed solution of paraformaldehyde (0.35 g, 11.5 mmol) and **2** (3.37 g, 11.5 mmol) in 1,2-dichloroethane (100 mL) under nitrogen atmosphere. Then the mixture was stirred at room temperature for 3 hour. A green solution was got. The reaction mixture was then washed with water (3 × 120 mL) and dried with excess $Na_2SO_4$. After the solvent was removed, the obtained solid was purified by column chromatography on silica gel with petroleum ether/dichloromethane (1:2 *v*/*v*) as the eluent to get a white powder of **3** (1.46 g, 45 %). The $^{1}$H NMR spectrum of **3** is shown in Figure S3. $^{1}$H NMR (500 MHz, $CDCl_3$, rt) $\delta$ (ppm): 6.915 (s, 10H), 4.229 (t, $J$ = 6.0 Hz, 20H), 3.850 (s, 10H), 3.630 (t, $J$ = 6.0 Hz, 20H). The $^{13}$C NMR spectrum of **3** is shown in Figure S4. $^{13}$C NMR (125 MHz, $CDCl_3$, rt) $\delta$ (ppm): 150.05, 129.45, 116.49, 69.35, 31.06, 29.77.

**Synthesis of CP5:** The mixture of compound **3** (1.5 g, 1.2 mmol) and trimethylamine (33 % in ethanol, 6.43 mL, 23.8 mmol) in ethanol (50 mL) were stirred at 90 °C for 24 h under nitrogen atmosphere. Then the solvent was removed by evaporation, deionized water (25 mL) was added. After filtration, a clear solution was got. Then the water was removed by evaporation to obtain **CP5** as a colorless solid (1.58 g, 95 %). The $^{1}$H NMR spectrum of **CP5** is shown in Figure S5. $^{1}$H NMR (400 MHz, $D_2O$, rt) $\delta$ (ppm): 6.909 (s, 10H), 4.412 (s, 20H), 3.881 (s, 10H), 3.765 (s, 20H), 3.167 (s, 90H). The $^{13}$C NMR spectrum of **CP5** is shown in Figure S6. $^{13}$C NMR (100 MHz, $D_2O$, rt) $\delta$ (ppm): 149.34, 129.89, 116.45, 64.88, 63.45, 54.04, 29.51.

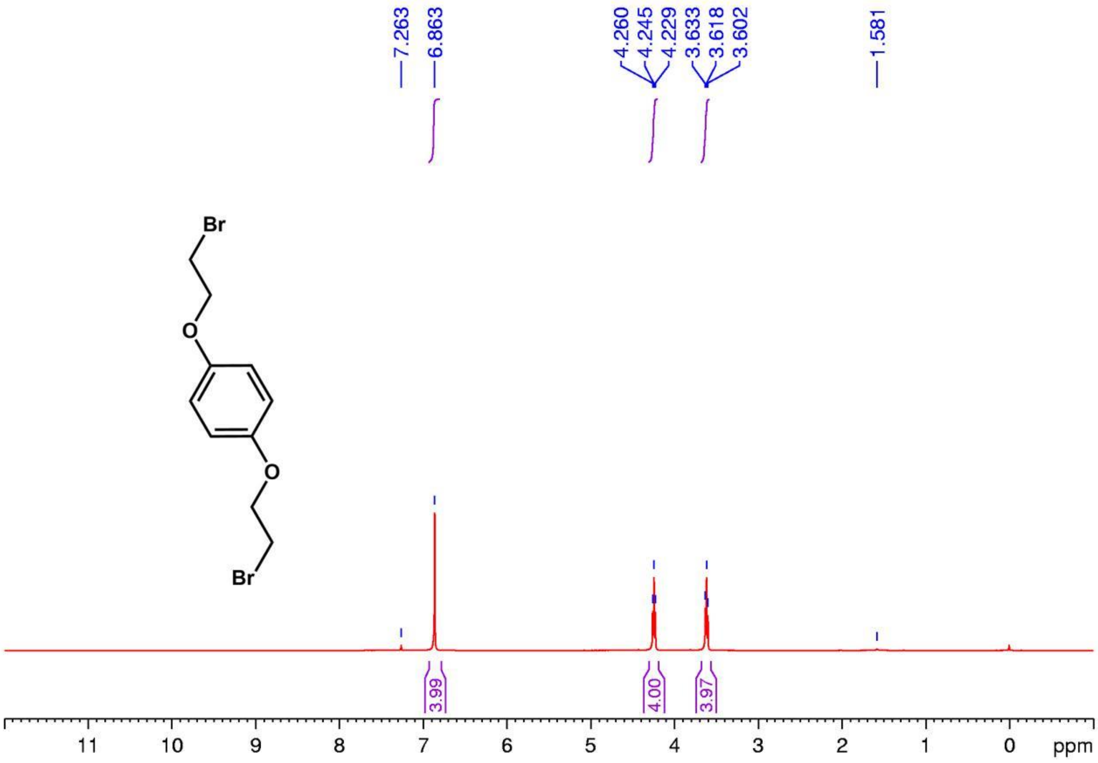


***Fig. S1*** $^{1}$H NMR spectrum (400 MHz, $D_2O$, 298 K) of **2**.

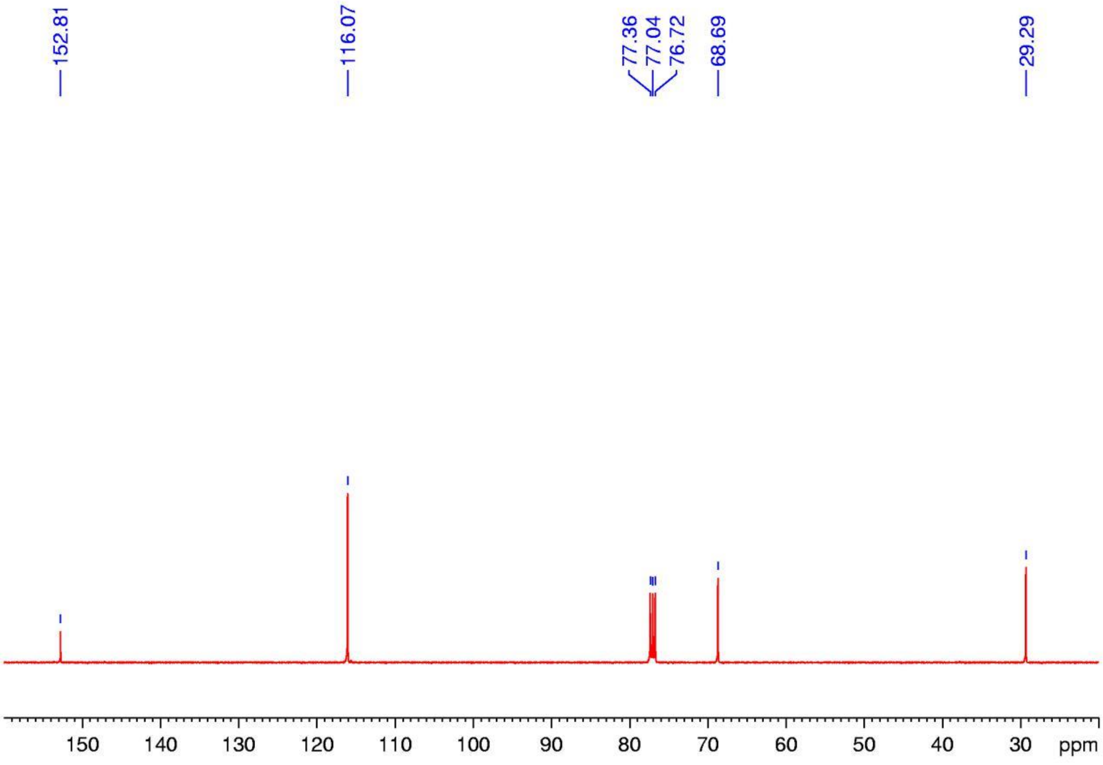


***Fig. S2*** $^{13}$C NMR spectrum (100 MHz, $D_2O$, 298 K) of **2**.

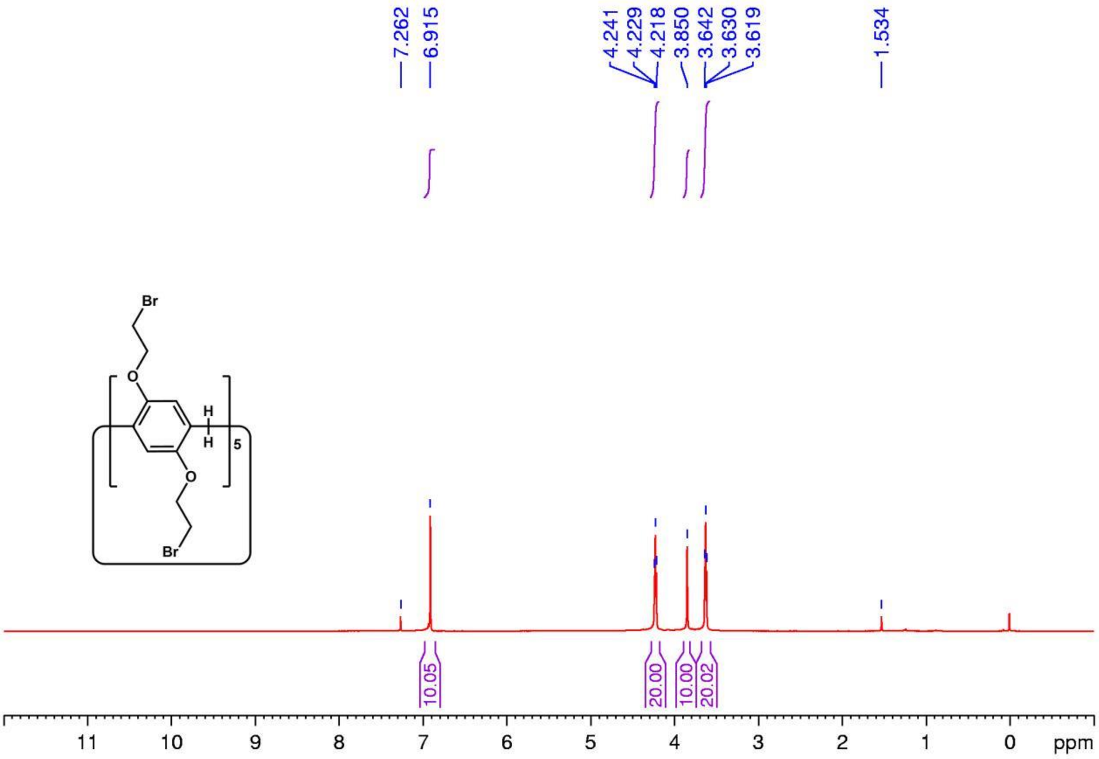


***Fig. S3*** ¹H NMR spectrum (500 MHz, $CDCl_3$, 298 K) of **3**.

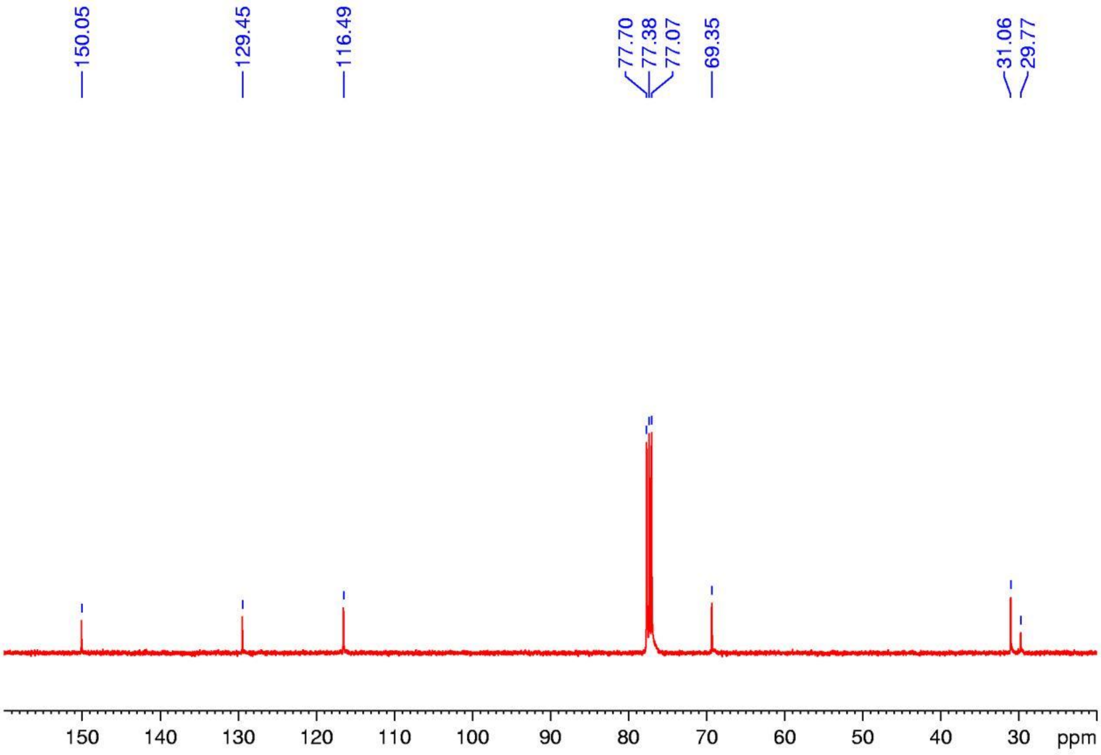


***Fig. S4*** ¹³C NMR spectrum (125 MHz, $CDCl_3$, 298 K) of **3**.

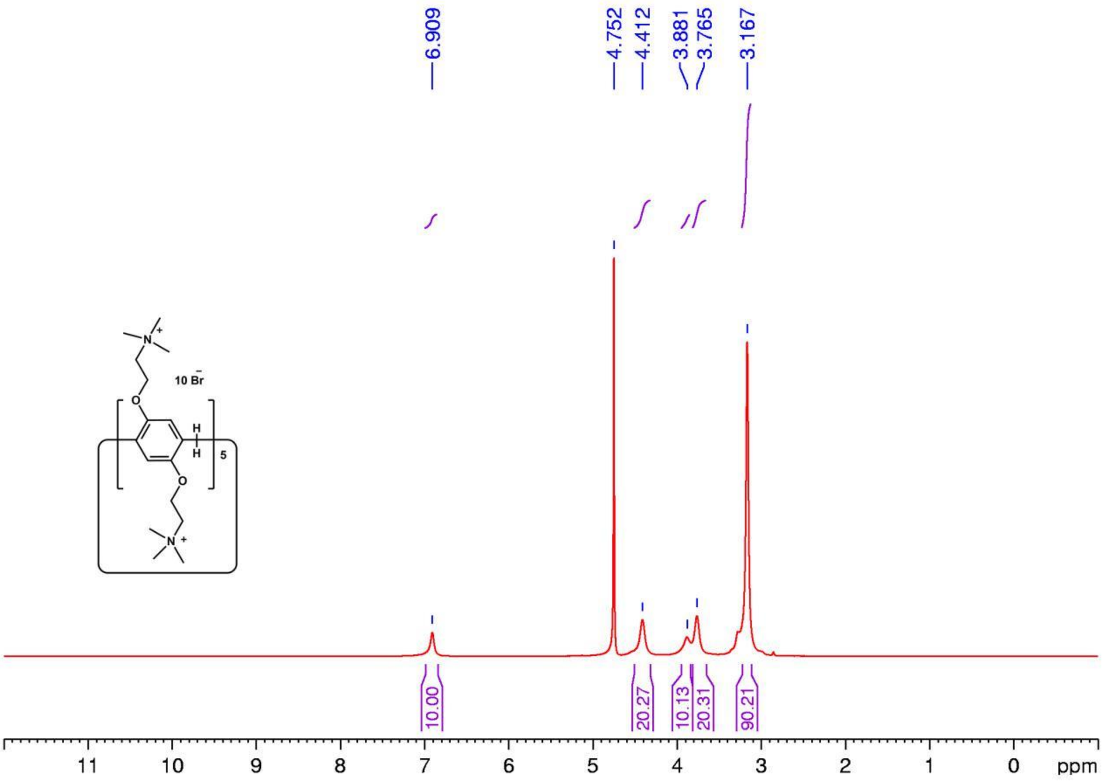


***Fig. S5*** ¹H NMR spectrum (400 MHz, $D_2O$, 298 K) of **CP5**.

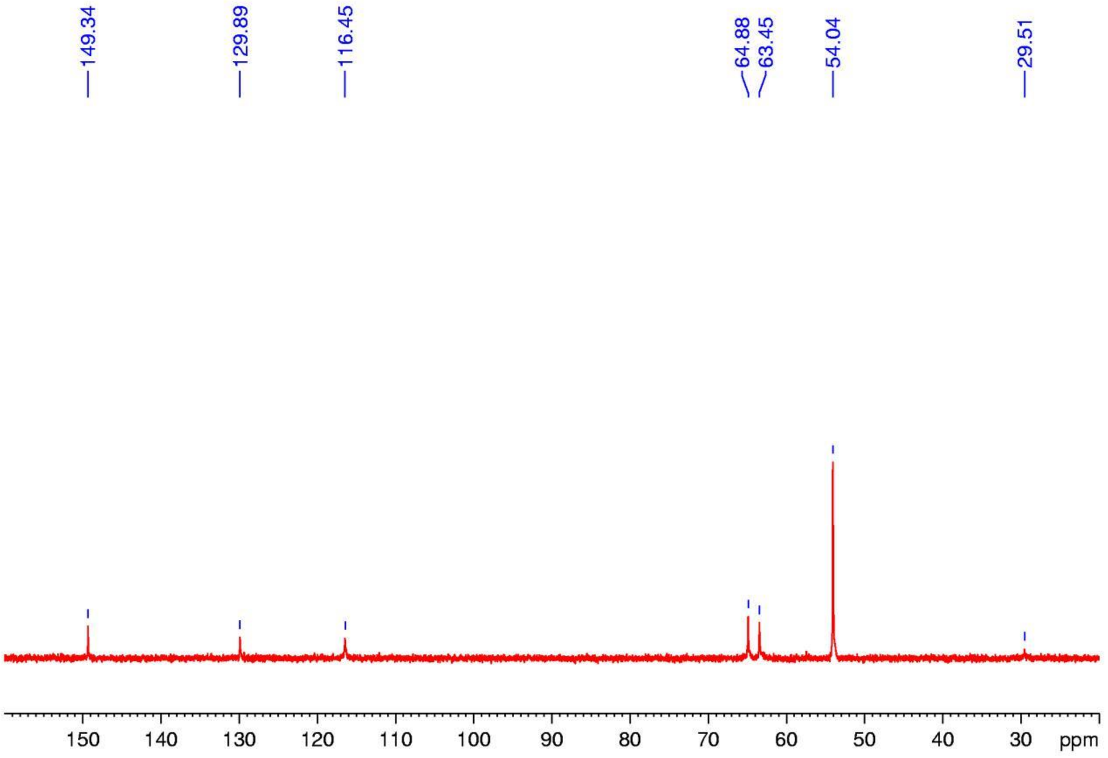


***Fig. S6*** ¹³C NMR spectrum (100 MHz, $D_2O$, 298 K) of **CP5**.

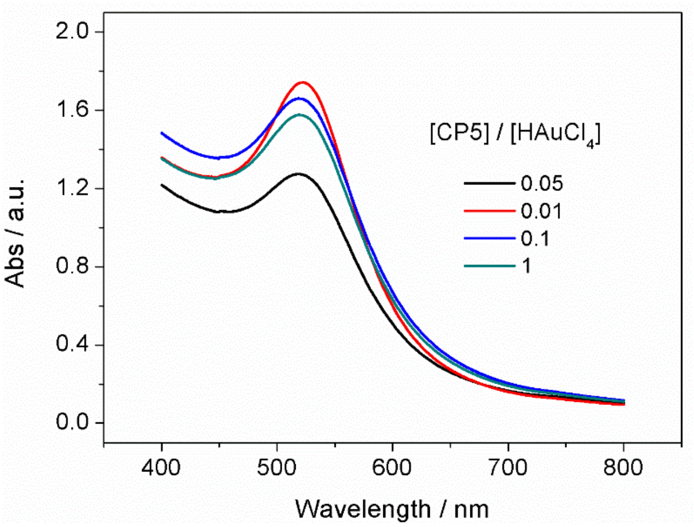


***Fig. S7*** Ultraviolet−visible spectra of CP5-modified Au-NPs synthesized with different [CP5]/[$HAuCl_4$] ratios.

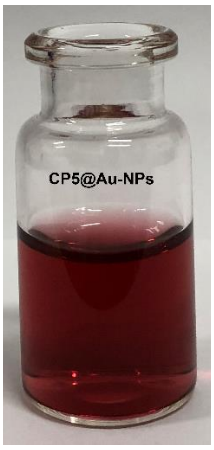


***Fig. S8*** Photograph for the aqueous solution of CP5@Au-NPs.

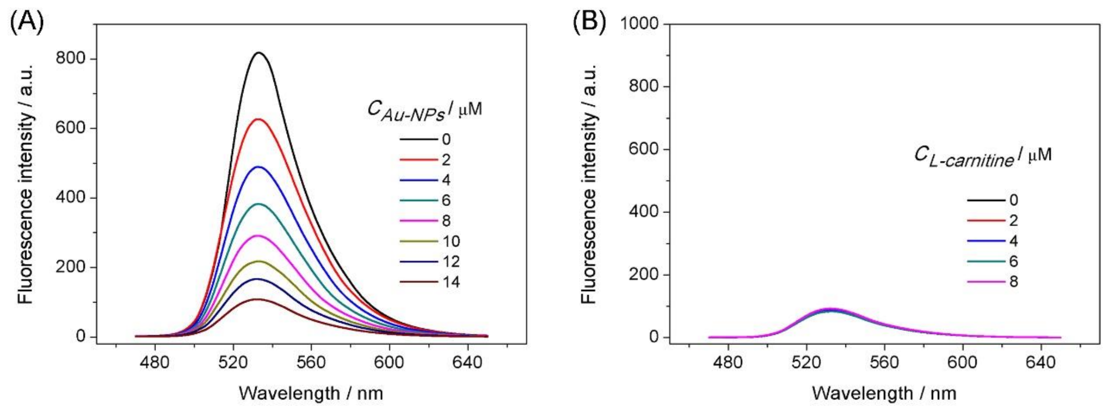


***Fig. S9*** The effect of increasing concentrations of Au-NPs (concentrations ranging from 0 to 14 μM) on the fluorescence intensity of 2 μM R123. (B) Fluorescence spectra of the R123@Au-NPs complex via different concentrations of L-carnitine.

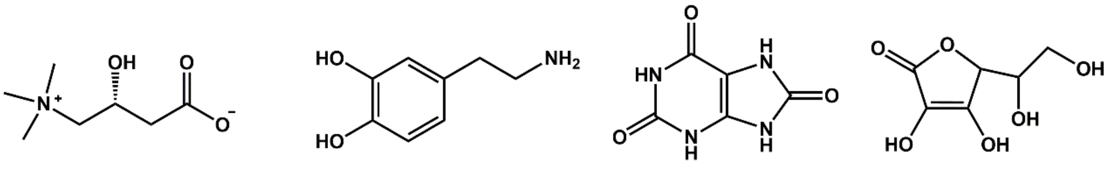


***Fig. S10*** Chemical structures of L-carnitine, DA, UA, and AA, respectively.

**Table S1** Comparison of different methods for quantitative detection of L-carnitine.

| Electrode or matrix | Method | Liner range (μM) | LOD (μM) | Ref |
|---|---|---|---|---|
| Ni/Cu alloy | Voltammetric | _ | 7.08 | 5 |
| CdSe/ZnS quantum dots | Fluorescence | _ | 0.15 | 6 |
| Cys−Au(I) | Fluorescence | 1-10 | 0.5 | 7 |
| Coenzyme A | Fluorescence | 0.5-10 | 0.18 | 8 |
| _ | Capillary zone electrophoresis | 9.1-200 | 3 | 9 |
| CMG/safranine T | Fluorescence | 0-534 | _ | 10 |
| PP5@Au-NPs | Fluorescence | 0.1-25 | 0.067 | This work |

**References**


1. Sanner, M.F. Python: a programming language for software integration and development. *J. Mol. Graph. Model*. **1999**, *17*, 57–61.
2. Maier, J.A.; Martinez, C.; Kasavajhala, K.; Wickstrom, L.; Hauser, K.E.; Simmerling, C. ff14SB: Improving the accuracy of protein side chain and backbone parameters from ff99SB. *J. Chem. Theory Comput*. **2015**, *11*, 3696–3713.
3. Ma, Y.; Ji, X.; Xiang, F.; Chi, X.; Han, C.; He, J.; Abliz, Z.; Chen, W.; Huang, F. A cationic water-soluble pillar[5]arene: synthesis and host-guest complexation with sodium 1-octanesulfonate. *Chem. Commun*. **2011**, *47*, 12340–12342.
4. Joseph, R.; Naugolny, A.; Feldman, M.; Herzog, I. M.; Fridman, M.; Cohen, Y. Cationic pillararenes potently inhibit biofilm formation without affecting bacterial growth and viability. *J. Am. Chem. Soc*. **2016**, *138*, 754–757.
5. Dehdari Vais, R.; Yadegari, H.; Sattarahmady, N.; Helia, H. An anodized nanostructure of Ni/Cu alloy synthesized in ethaline for electrocatalytic oxidation and amperometric determination of L-carnitine. *J. Electroanal. Chem* **2018**, *815*, 134–142.
6. Li, H. B.; Zhang, Y.; Wang, X. Q. L-Carnitine capped quantum dots as luminescent probes for cadmium ions. *Sensor Actuat B* **2007**, *127*, 593–597.
7. Tseng, Y.T.; Chang, H.Y.; Harroun, S.G.; Wu, C.W.; Wei, S.C.; Yuan, Z.Q.; Chou, H.L.; Chen, C.H.; Huang, C.C.; Chang, H.T. Self-assembled chiral gold supramolecules with efficient laser absorption for enantiospecific recognition of carnitine. *Anal. Chem*. **2018**, *90*, 7283−7291.
8. Wang, M.H.; Gu, J.A.; Mani, V.; Wu, Y.C.; Lin, Y.J.; Chia, Y.M.; Huang, S.T. A rapid fluorescence detecting platform: applicable to sense carnitine and chloramphenicol in food samples. *RSC Adv*. **2014**, *4*, 64112–64118.
9. Kong, Y.; Yang, G.F.; Chen, S.M.; Hou, Z.W.; Du, X.M.; Li, H.; Kong, L.H. Rapid and sensitive determination of L-carnitine and acetyl-L-carnitine in liquid milk samples with capillary zone electrophoresis using indirect UV detection. *Food Anal. Methods* **2018**, *11*, 170–177.
10. Mao, X.W.; Tian, D.M.; Li, H.B. p-Sulfonated calix[6]arene modified graphene as

a ‘turn on’ fluorescent probe for L-carnitine in living cellsw. *Chem. Commun*. **2012**, *48*, 4851–4853.
